# A Diagnosis of Adult-Onset Still's Disease after Multiple Urgent Care Visits

**DOI:** 10.1155/2019/6245158

**Published:** 2019-09-10

**Authors:** Kami M. Hu, Adam C. Richardson, Kelly M. Blosser, Semhar Z. Tewelde

**Affiliations:** ^1^University of Maryland School of Medicine, Department of Emergency Medicine, Baltimore, MD, USA; ^2^University of Maryland Medical Center, Department of Emergency Medicine, Baltimore, MD, USA

## Abstract

A 34-year-old man with recent treatment and resolution of community-acquired pneumonia presents to the emergency department with protracted fever, rash, and sore throat. Sustained fever and greater than two-fold increase in leukocytosis despite appropriate antibiotic therapy prompted hospital admission for infectious disease and rheumatologic evaluations which ultimately revealed adult-onset Still's disease, a rare autoinflammatory disorder with potentially life-threatening complications.

## 1. Introduction

Adult-onset Still's disease (AOSD) is rare, with unknown true incidence likely secondary to underdiagnosis and the insidious nature of its presentation. Untreated AOSD can result in life-threatening complications including macrophage activating syndrome (MAS) and disseminated intravascular coagulation (DIC) [1]. Treatment can be relatively straightforward with corticosteroids, but success is dependent on early diagnosis and course [2]. Providers working in emergency medicine and primary care should be familiar with common variations of symptomatology and progression as early signs are often quite vague.

## 2. Case Report

A 34-year-old male presented to the emergency department (ED) complaining of persistent fevers, chills, night sweats, generalized body aches, a dry cough, odynophagia, and a mild palpable rash on his bilateral clavicles and right thigh. Approximately ten days prior he presented to a local urgent care with similar complaints, and a chest X-ray revealed infiltrates in the left lower lobe and the retrocardiac region. At that time his white blood cell count (WBC) was noted to be 13 × 10^9^ cells/mm^3^. The diagnosis of community-acquired pneumonia was made and he completed a course of azithromycin. Symptom resolution lasted less than one week and recurrence prompted him to return to the urgent care clinic. A repeat chest X-ray revealed resolution of previous infiltrates; however, the complete blood count revealed a leukocytosis of 29 × 10^9^ cells/mm^3^ with predominant granulocytes. He was subsequently advised to present to the ED.

In the ED he was most concerned about his sore throat as well as a feeling of obstruction in his throat with continual fevers. He also noted a nonpruritic rash on his clavicles and thigh, but rated these symptoms as minor in comparison. On examination his triage vital signs were all within normal limits, oropharynx was mildly erythematous without exudates, and breath sounds were clear to auscultation. There was a fine, palpable, mildly erythematous rash noted over the region of his clavicles bilaterally. Our complete blood count was consistent with that of the urgent care. A comprehensive metabolic panel demonstrated a transaminitis with an aspartate aminotransferase of 108 units/L, alanine aminotransferase of 205 units/L, total bilirubin of 1.7 mg/dL, direct bilirubin of 1.2 mg/dL, and alkaline phosphatase of 734 units/L. An initial lactic acid of 1.8 mmol/L normalized after one liter of crystalloids and blood work was otherwise within normal limits. Contrast-enhanced computed tomography scan of the soft tissue of the neck and chest were obtained to rule out retropharyngeal abscess or occult pneumonia, but no acute abnormalities were discovered.

The patient developed a fever of 39 degrees Celsius during the ED encounter and was admitted to the internal medicine team for fever of unknown origin. Blood cultures were obtained but did not result in growth of bacteria. A rheumatology consult was completed during his admission and further laboratory testing revealed an erythrocyte sedimentation rate (ESR) greater than 120 mm/hr, a ferritin of 24,000 ng/mL, and a negative antinuclear antibody. The rheumatology team included AOSD on the differential diagnosis early and as such, the diagnosis was made promptly and corticosteroid therapy was initiated with 60 mg of daily prednisone. The patient reported dramatic improvement of symptoms within one week of initiating steroid therapy. The WBC, specifically the granulocytes, decreased by 30% after only one day of steroid therapy and continued to normalize over one week's time. The ESR, CRP, ferritin, lactate, and WBC were monitored and all trended down over the course of the first week of treatment. During outpatient rheumatology follow-up, prednisone was titrated down over the course of several months; one year later the patient was in remission, doing well off of corticosteroid therapy and was even able to run a marathon. The team wondered if the earlier community-acquired pneumonia was a triggering event for the occurrence of AOSD, but were ultimately unsure as the pneumonia resolved expeditiously with a course of azithromycin.

## 3. Discussion

AOSD is a rare, systemic autoinflammatory disorder thought to be related to systemic-onset juvenile idiopathic arthritis, with an underlying pathophysiology that remains unclear. It is estimated to occur in approximately 1 to 34 out of 1 million people, with a bimodal age distribution, occurring most often in the age ranges of 15–25 and 35–45 years, although diagnosis after the age of 60 has been documented [1]. AOSD remains a diagnosis of exclusion, and it is imperative that providers rule out infectious, neoplastic, and toxic syndromes before settling on the diagnosis. The initial presentation commonly includes vague symptoms such as recurrent fevers, rashes, arthralgias, pharyngitis, and lymphadenopathy. Characteristic lab findings include granulocytic leukocytosis, transaminitis, hyperferritinemia, and a negative antinuclear antibody and rheumatoid factor test. Some of these features are included in the Yamaguchi criteria [3], a set of major and minor criteria including clinical symptoms, exam findings, and lab tests (Table 1), with both a sensitivity and specificity of approximately 95% [4]. Diagnosis requires at least five of the Yamaguchi criteria, at least two of which must be major criteria, and the absence of infection, malignancy, or other rheumatologic diseases.

AOSD should be considered in any patient with fever of unexplained origin who prompts a rheumatologic and/or occult infectious workup, as early recognition and expeditious initiation of treatment are necessary in order to decrease morbidity and mortality as complications can include pulmonary arterial hypertension, coagulopathies, amyloidosis, fulminant hepatitis, macrophage activation syndrome (MAS), and disseminated intravascular coagulation (DIC) [5]. A 2013 review of those admitted with a diagnosis of AOSD in the nationwide inpatient sample database revealed an in hospital death rate of 2.8% and serious complications such as MAS and DIC at 2.4% [6]. Another tool available to clinicians that has proven useful in predicting poor outcomes is Pouchot's Systemic Score, which involves assigning one point to each of the twelve clinical manifestations including fever, typical rash, pleuritis, pneumonia, pericarditis, hepatomegaly or transaminitis, splenomegaly, lymphadenopathy, leukocytosis of at least 15 × 10^9^ cells/mm^3^, pharyngitis, myalgias, and abdominal pain [7]. A score of seven or higher in conjunction with a serious complication such as MAS, DIC, or renal failure is associated with higher mortality [8]. Our patient's score was eight, as he experienced fevers, rash, pneumonia, transaminitis, splenomegaly, lymphadenopathy, leukocytosis, and pharyngitis, but fortunately he did not experience a serious complication or a poor outcome.

With respect to therapy, randomized trials investigating the best treatment strategy for AOSD are lacking. While some experience a single systemic episode, termed “monocyclic” AOSD, it is estimated that two-thirds of patients diagnosed with AOSD go on to experience a “polycyclic” variant involving chronic, recurring episodes while up to 30% of patients with AOSD experience a severe form of the illness associated with higher morbidity and mortality [2]. The unclear pathophysiology and presentation's variance in symptomatology and severity leaves a variety of potential targets for treatment. Nonsteroidal anti-inflammatory drugs (NSAIDs) were once considered the appropriate initial treatment and remain on the list of potential therapies, but they have been demonstrated to be less effective at actually inducing remission and are associated with their own adverse events [2, 9]. Currently, glucocorticoids are generally accepted as first-line therapy for initial treatment of AOSD, although they are better at controlling systemic symptoms than articular ones and seem to work best for monocyclic AOSD, failing to induce remission in up to 40 percent of cases [2, 9, 10]. Prolonged steroid use is problematic due to the side effect profile, but for some patients there may be difficulty weaning without return of symptoms. The use of disease-modifying antirheumatic drugs (DMARDs) such as methotrexate or cyclosporine [2, 9, 11] and biologics such as interleukin antagonists have shown efficacy as steroid-sparing agents, treatment in steroid-refractory AOSD, and in some cases, as first-line agents themselves with the specific interleukin-targeted depending on whether the AOSD presentation is more systemic (IL-1) or articular in nature (TNF-alpha, IL-6) [2, 9, 10]. In more severe presentations of AOSD, including DIC or MAS, immediate initiation of a biologic, or even intravenous immunoglobulin (IVIG) in addition to pulse dose steroids is recommended [2, 10].

## 4. Conclusion

Emergency medicine providers are not likely to make the official diagnosis of AOSD, but should be aware of the Yamaguchi criteria when considering differential diagnoses in relevant patients. Our patient was initially thought to most likely have a viral syndrome, but his impressive leukocytosis and fever of unknown origin prompted admission and fortunately rheumatologic consultation. His diagnosis was made early and he had a good outcome, avoiding the serious complications that can accompany a delayed diagnosis.

## Figures and Tables

**Table 1:** 

Major criteria	Minor criteria
Fever over 39°C > one week	Pharyngitis
Arthralgias > two weeks	Transaminitis
Nonpruritic rash (typically salmon-colored) while febrile	Lymphadenopathy
Predominantly granulocytic leukocytosis at least 10 × 10^9^ cells/mm^3^	Hepatomegaly and/or splenomegaly
The Yamaguchi criteria [3].	Negative antinuclear antibody and rheumatoid factor tests
